# Mucin-Type *O-*Glycosylation in Invertebrates

**DOI:** 10.3390/molecules200610622

**Published:** 2015-06-09

**Authors:** Erika Staudacher

**Affiliations:** Department of Chemistry, University of Natural Resources and Life Sciences, Vienna, Muthgasse 18, 1190 Vienna, Austria; E-Mail: erika.staudacher@boku.ac.at; Tel.: +43-1-47654 (ext. 6063); Fax: +43-1-47654-6059

**Keywords:** *O-*glycosylation, invertebrates, mucin-type, nematode, insect, helminth, mollusc

## Abstract

*O-*Glycosylation is one of the most important posttranslational modifications of proteins. It takes part in protein conformation, protein sorting, developmental processes and the modulation of enzymatic activities. In vertebrates, the basics of the biosynthetic pathway of *O-*glycans are already well understood. However, the regulation of the processes and the molecular aspects of defects, especially in correlation with cancer or developmental abnormalities, are still under investigation. The knowledge of the correlating invertebrate systems and evolutionary aspects of these highly conserved biosynthetic events may help improve the understanding of the regulatory factors of this pathway. Invertebrates display a broad spectrum of glycosylation varieties, providing an enormous potential for glycan modifications which may be used for the design of new pharmaceutically active substances. Here, overviews of the present knowledge of invertebrate mucin-type *O-*glycan structures and the currently identified enzymes responsible for the biosynthesis of these oligosaccharides are presented, and the few data dealing with functional aspects of *O-*glycans are summarised.

## 1. Introduction

Protein glycosylation plays an important role in several types of recognition processes ranging from fertilisation and development to pathological events and cell death. 30 years ago, the role of *O-*glycans was only seen in the influence of physical parameters, such as ensuring protein stability and tertiary structure, providing the basis for rigid conformations and slowing down proteolytic degradation of the peptide chain; most of specialised recognition events have been allocated to *N*-glycans. However, it is now clear that different types of *O-*glycans are relevant for many more functions, such as modulating of enzyme activity (e.g., the reversible attachment of an *O-*linked GlcNAc (*N*-acetylglucosamine) residue to cytoplasmic and nuclear proteins), modulating pro-protein processing and acting as signal molecules or sorting determinants guiding the modified protein in the cell from the place of biosynthesis to its target location [1,2,3,4]. The functional and pathological consequences of altered *O*-glycans in human cancer have been determined [5,6]. *O*-glycans are essential in the von Willebrand factor [7], critical for vascular integrity [8], regulating secretion and secretory vesicle formation [9] and are essential during eukaryotic development [10].

In *N*-glycans, the linkage of a glycan to the protein is always formed by a GlcNAc-residue linked to an asparagine within the consensus sequence Asn-Xxx-Ser/Thr. Biosynthesis begins in the endoplasmic reticulum by forming a highly conserved oligosaccharide structure (two GlcNAc, nine Man (mannose), three Glc (glucose) residues) on a lipid precursor. Then, the glycan is transferred *en bloc* onto the growing peptide chain and is maturated within Golgi vesicles. In *O-*glycans, in contrast, a number of amino acids (serine, threonine, tyrosine, hydroxylysine and hydroxyproline) can be connected with several involved sugar-residues (GalNAc (*N*-acetylgalactosamine), GlcNAc, Man, Glc, Gal (galactose), Ara (arabinose), Xyl (xylose), Fuc (fucose) and *N*-acetylfucosamine, with no fixed consensus sequence [11]. *O-*Glycan biosynthesis begins by the transfer of one sugar to the protein, followed by the addition of more monosaccharides one by one. Furthermore, the first step of biosynthesis is not restricted to a single organelle but may occur, depending on the linkage formed, in the endoplasmic reticulum, Golgi apparatus, cytosol or nucleus [1]. Most structural investigations on *O-*glycans have been carried out on mucin-type *O-*glycans (GalNAc-α-Ser/Thr), which frequently occur in higher eukaryotes. Mucin-type *O-*glycosylation is initiated by the transfer of a GalNAc residue from UDP-GalNAc to a Ser or Thr of a polypeptide chain (for enzyme classification see [12]). Eight core subtypes with different tissue expression patterns have been described in mammals (reviewed in [13]). Depending on the organism, tissue and developmental stage, these cores are elongated and modified by GlcNAc, Gal and Fuc, and are often sialylated and sometimes sulphated. The direct peptide-linkage of monosaccharides other than GalNAc monosaccharides is characteristic for other types of *O-*glycosylation. A GlcNAc-β-Ser/Thr without any elongation is a typical feature of signal recognition for nuclear and cytoskeletal proteins. In many cases, *O-*GlcNAcylation competes with phosphorylation [14]. A protein-linked α-Fuc residue with or without elongation has been found to be essential in epidermal growth factor domains, together with Glc-β-Ser [1]. The biosynthesis of chondroitin and heparan sulphates, large glycan chains with disaccharide repeating units, is initiated by *O-*xylosylation of the peptide. *O-*Mannosylation is a typical yeast feature, but it has also been found in some muscular dystrophies [15].

**Figure 1:**
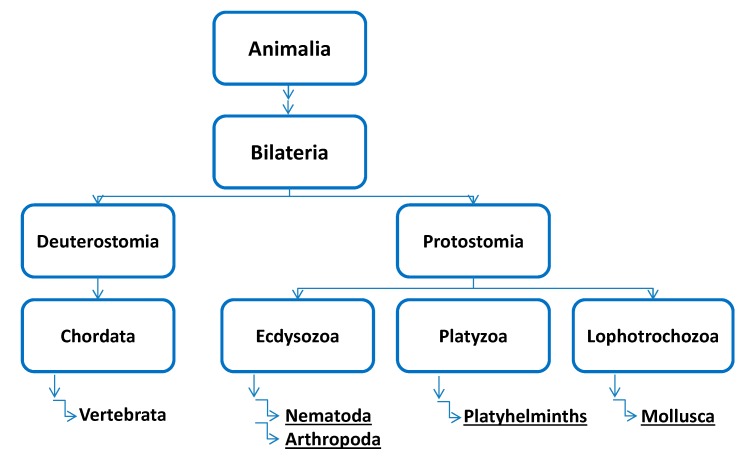
Evolution tree of the kingdom of *Animalia* (condensed). Only those phyla where at least some information about *O-*glycans is available are given. Underlined phyla are topic of this review.

Particularly for invertebrates the knowledge regarding any kind of *O-*glycosylation is very rudimentary (Figure 1). Even when some structural aspects are solved, the corresponding biosynthetic active enzymes are often not identified or characterized. This limits the opportunities for determining structure-function relations. However, the few existing data lead to the suggestion that a broad spectrum of modifications and recognition events are still waiting to be discovered. This review aims to motivate further reading and stimulate the interest in invertebrates.

Here, the focus will be on the current knowledge of mucin-type *O-*glycosylation in invertebrates mainly covering structural aspects as well as the corresponding enzymes. Investigations on other types of *O-*glycosylation in invertebrates are currently restricted to a few model organisms (*Drosophila melanogaster* and *Caenorhabditis elegans*) and have been discussed recently (for *O-*GlcNAc see [16], for *O-*Fuc see [17], for *O-*Glc see [18], for *O-*Man (mannose) see [19,20] and for xylosyltransferases initiating proteoglycans see [21]).

## 2. Nematoda

**Figure 2:**
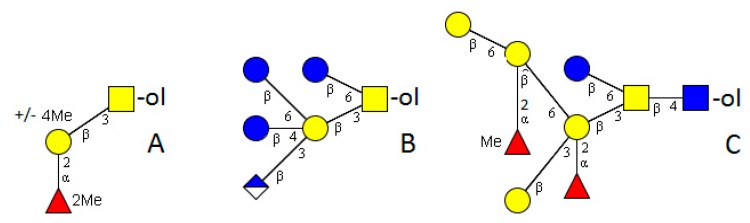
Examples of extended *O-*glycans in nematodes. (**A**) *Toxocara* [22]; (**B**,**C**) *Caenorhabditis elegans* [26]. The structure plots (Figure 2, Figure 3, Figure 4, Figure 5 and Figure 6) were generated in the notation of the Consortium for Functional Glycomics (http://www.functional glycomics.org) using the visual editor of “GlycoWorkbench”. This software application was developed as part of the EUROCarbDB project and is available online (http://www.ebi.ac.uk/pdbe-srv/eurocarbdb/). Yellow square = GalNAc, blue square = GlcNAc, yellow circle = Gal, blue circle = Glc, red triangle = fucose, upper blue diamond = GlcA (glucuronic acid).

Studies on *Toxocara* excretory-secretory larvae antigens determined for *T. canis* (a parasite of dogs and canid animals) 2-*O-*Me-Fucα1-2(4-*O-*Me)Galβ1-3GalNAc and 2-*O-*Me-Fucα1-2Galβ1-3GalNAc, both based on core 1 type structures, as major oligosaccharides (Figure 2), while in the corresponding parasite for cats, *T. cati*, only the di-*O-*methylated trisaccharide was present [22]. Both are targets of the strong immune response against *Toxocara* excretory-secretory antigens [23].

For *Trichinella spiralis* highly immunogenic characteristic tetra-antennary tyvelose containing *N*-glycans have been described [24]. An antibody reaction against an *O-*glycan/peptide was detected, but no detailed sugar structure was determined in this case [25]. No *O-*glycan structures were found in a detailed investigation of the intestinal parasitic nematode of cattle, *Cooperia oncophora* [26].

The worm model organism *Caenorhabditis elegans* is rather well studied. A number of unusual mucin-type structures have been found in an extensive NMR-based study. Based on core 1 type (Galβ1-3GalNAc) or the “invertebrate core” type (Galβ1-6(Galβ1-3)GalNAc), a series of structures containing GlcA and β1-4- and/or β1-6-linked Glc residues were detected (Figure 2). The Glc residues on mucin-type *O-*glycans and the extension of GalNAc by three sugars are unique. Furthermore, a large branched structure with GlcNAc instead of GalNAc directly linked to the protein was identified. This structure contained α1–2 linked Fuc with and without an additional methyl group in terminal position (Figure 2) [27]. In 1996, an indication of the presence of phosphocholine on *O-*glycans of a nematode of *Onchocerca gibson*, a parasite of Australian cattle, was found but no detailed structure was determined [28].

At least nine distinct members of the UDP-GalNAc:polypeptide GalNAc transferase family [GalNAc-T, E.C. 2.4.1.41] have been identified in *C. elegans*. They are highly conserved type-2 membrane proteins with a 60%–80% sequence identity to their human counterparts [29]. They fit well into the phylogenetic subfamilies of the putative orthologous genes of mammals and flies according to their structure, their acceptor specificity and their kinetic data. Their expression patterns vary significantly in the different tissues of *C. elegans* [30]. These facts support the hypothesis that important biological functions are associated with specific isoforms [31]. The subsequent enzyme forming core 1 type *O-*glycan structures is a β1-3-galactosyltransferase [β1-3-Gal-T, T-synthase, E.C. 2.4.1.122], which transfers Gal from UDP-Gal to GalNAcα1-Ser/Thr. The *C. elegans* enzyme consists of 389 amino acids with an overall identity of 42.7% to human T-synthase. In contrast to the human enzyme, the worm enzyme contains four putative *N*-glycosylation sites and does not require Cosmc (“core 1 βGal-T specific molecular chaperone”) for activity [32]. Furthermore, an α1-3-Fuc-T family [33] and an UDP-GalNAc:GlcNAcβ-R β1-4-*N*-acetylgalactosaminyltransferase [34] were cloned and characterized, but it is not completely clear whether they act *in vivo* as modifier of the termini of *N*- or of *O-*glycans, or both.

Thus far, the function of mucin-type *O-*glycosylation in *C. elegans* is not clear in detail. But RNAi studies of those genes involved in glycosylation pathways showed that there is an unique requirement for proteoglycan modification of the cell surface for the terminal phase of cytokinesis in early *C*. *elegans* embryos [35,36].

## 3. Arthropoda

### 3.1. Crustacea (Crustaceans)

A putative *O-*glycosylation site has been identified in the sequence of a catechol-*O-*methyltransferase from the shrimp *Penaeus monodon*, which seems to play a role in salinity stress tolerance under low salinity conditions [37]. In the hemocyanin of the crab *Carcinus aestuarii*, three *O-*glycosylated tripeptides were identified by mass spectrometry and subsequent exoglycosidase digests. Each glycopeptide consisted of two *O-*linked glycan chains, where the binding sites were separated by one amino acid. In the first peptide, two *O-*GalNAc residues were linked to a Ser and a Thr, while all other glycans were linked exclusively to Ser (GalNAc-residues elongated by Gal and *N*-Acetyl-*O-*NeuAc or *N*-Acetyl-*O-*di-NeuAc) [38].

### 3.2. Hexapoda (Insects)

In relation to the enormous biodiversity of insects, our knowledge on insect glycosylation is rather limited as funding is often only available if analysis is somehow connected with human nutrition or health (*i.e.*, pathogens, allergens or poisons). Therefore, insect glycosylation has been investigated only for the model organism *Drosophila melanogaster*, some cell lines used for expression and some venoms which are relevant to allergies.

#### 3.2.1. Drosophila Melanogaster

**Figure 3:**
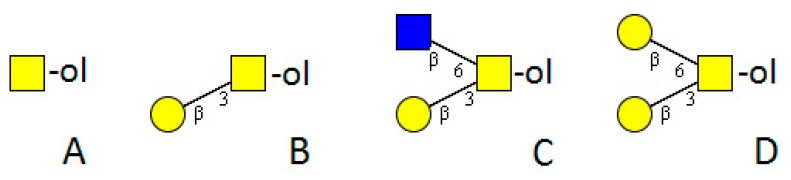
Basic core structures common in invertebrates. (**A**) Tn-antigen; (**B**) T-antigen; core 1 type structure; (**C**) core 2 type structure; (**D**) invertebrate core.

**Figure 4:**
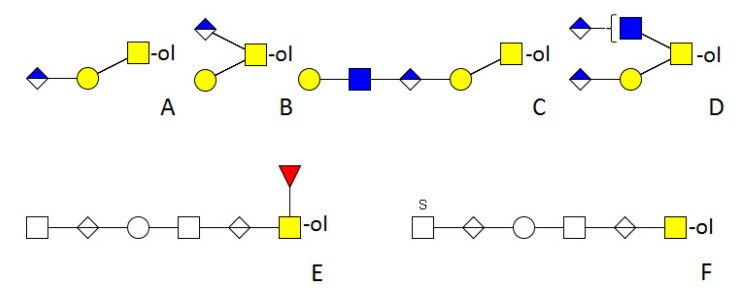
Examples of extended *O-*glycans in insects. (**A**–**D**) *Drosophila melanogaster* [41,42]; (**E**,**F**) were derived from a recombinant protein produced in High-Five™ cells [61]. Empty square = *N*-acetylhexosamine, empty circle = hexose, empty diamond = uronic acid, S = sulphate.

*Drosophila melanogaster* is the model organism for insects. Its genome is completely sequenced and the posttranslational modifications are well investigated [39]. Due to the well-established *Drosophila*-system in several laboratories, much is known about the function of its different structures and related enzymes. Core 1 type is the dominant *O-*glycan structure [40]. It can be modified by GlcNAc forming core 2 type structures (Figure 3). Further elongations by GlcA linked to GalNAc or a Man residue are possible. GlcA may be in a terminal or internal position and is attached to any type of core. Some cores with more than one GlcA are present (Figure 4) [41]. No modification by phosphoethanolamine or sulfation has been found [42]. A rather unusual trisaccharide with an *O-*linked Fuc extended by β1-3GlcNAc and β1-4GlcA comprised 11.3% of the total *O-*linked glycan profile in wild-type embryo powder, but its precursors *O-*Fuc or GlcNAcβ1-3Fuc were not determined [42]. Surprisingly, Fuc was not found in any other structure.

Corresponding to vertebrates in *Drosophila,* an evolutionary conserved family of GalNAc-Ts initiating the mucin-type *O-*glycosylation has been found (10 biochemically confirmed genes, four putative members of the family). The members are expressed in different tissues and temporal patterns during development and organogenesis and have been shown to be essential for viability [31,43,44,45,46,47]. The enzymes have high sequence identity to the mammalian enzymes and display similar acceptor preferences [48]. β1-3-Gal-Ts, necessary for forming the core 1 type structure (Figure 3), have also been identified, and one was confirmed as essential for neural development [49,50]. Overexpression of Fuc-TA, an α1-3-Fuc-T acting on *N*-glycans, affects Notch signalling. Presumably this is due to the rendered *O-*fucosylation caused by imbalanced GDP-Fuc consumption [51]. Two specific Xyl-Ts transferring Xyl into α1,3-linkage towards *O-*linked Glc have been identified [52]. The rather striking variety of glycosylation in *Drosophila* seems to be controlled to some extent by a fragmentation of the Golgi into functional units with different enzyme equipment and transport opportunities [53].

*Drosophila melanogaster* is one of the few organisms in which the function of *O-*glycosylation has been investigated in detail. First, *O-*glycosylation-dependent changes in epithelial tube formation were detected [54], and later, the relatively new method of RNA interference allowed for the determination of several more functional and morphological changes. Reduced expression of individual GalNAc-T genes resulted in reduced secretion, altered epithelial cell adhesion and cytokinesis or changes in Golgi organisation, thus confirming the importance of *O-*glycosylation [55,56]. Normal muscle development requires *O-*mannosylation of *Drosophila* *dystro-glycan* homologue [20].

#### 3.2.2. Venoms

The N-glycans of some insect venoms became of interest because of their involvement in allergic reactions [57], but no attention was paid to *O-*glycans. Just in the nests of *Vespula germanica*, Tn and T-antigen *O-*glycans were determined along with their derivatives modified by 2-aminoethyl phosphate (Figure 3) [58].

#### 3.2.3. Insect Expression Systems

Insect cells are efficient tools for the expression of recombinant glycoproteins. There are several cell lines established from moth (*Lepidoptera*) origin. The most frequently used are derived from the fall armyworm *Spodoptera frugiperda* (Sf9, Sf21) and the cabbage looper *Trichoplusia ni* (High Five™); Others are from the silkworm *Bombyx mori*, the cabbage moth *Mamestra brassicae* or the Acrea moth *Estigmene acrea*. Besides technical requirements enabling large-scale protein production, such as growing of the cells, baculovirus infection rate, expression rate and downstream processing, the expression system is selected due to its ability to form the desired protein as similarly as possible to the mammalian model. Correct glycosylation is a main factor in this attempt. Therefore, the glycosylation potential of these cell lines was investigated to analyse their original abilities and to evaluate the engineering needs in order to mimic the glycosylation patterns of mammals.

All investigated cell lines have the ability to form mucin-type *O-*glycosylation. However, the structures differ with regard to cell line and medium. Tn-antigens were detected, the activities of more than one GalNAc-T activity could be determined, and the formation of core 1 type structures, including the activity of the responsible enzymes, was shown [59]. A further elongation of this core by an α1-4-linked Gal residue was observed, and the enzyme was characterised [60]. Mass spectrometric analysis and lectin detection revealed that the recombinant expressed glycans were generally much larger and more diverse than the native ones in a previous study. Large extensions containing several HexNAc and HexA residues were found. Fuc directly linked to the protein and extended by uronic acid and HexNAc (HexNAc-HexA-Fuc-ol) or as an extension of the core (HexA-(Fuc)-GalNAc-ol) was also detected (Figure 4) [61].

A fascinating issue is the question of sialylation in insects. There are a number of controversial studies regarding the ability of sialylation in insects. In *Drosophila, in situ* hybridization analysis of embryonic expression revealed a very restricted pattern of *Drosophila* sialyltransferase in a few cells of the developing central nervous system [40]. Furthermore, the cytosine-monophosphate-sialic acid synthetase which provides the donor substrate, as well as the sialyltransferase gene, were found in the central nervous system cells [62,63]. In lepidopteran insect cell lines, no sialyltransferase activity or terminal sialylation was detected [59]. The negative charge related to the *O-*glycans of recombinant expressed proteins generated in Sf9 and High Five™ cells was determined to be GlcA, GalA, sulphate and phosphocholine [61]. Sialylation in insects is thus restricted to few tissues and some developmental stages and is therefore not as common as in mammals.

Even when the moth expression systems and the model organism *Drosophila melanogaster*, in particular, are well investigated, we cannot claim to know much about insects. Thus far, about one million species are zoologically described, but about five times more are anticipated. Currently, we only have information from a handful of species regarding their glycosylation abilities.

## 4. Platyhelminthes

A number of helminth parasites show *O-*glycan structures and mucins. In particular, the Tn antigen is widespread. The parasitic flatworm *Fasciola hepatica*, a trematode which causes fasciolosis, a chronic disease in humans and domestic animals, expresses Tn (mainly in the testis) as along with sialyl-Tn-antigens (widely distributed in different tissues), which may play a role in the interaction with the host in terms of mimicry or influence on the immune system (Figure 3) [64]. The Tn antigen was also found in *Echinococcus granulosus*, the sheep-dog tapeworm [65]. In both *F. hepatica* and *E. granulosus*, high activity levels of the *O-*glycan initiating enzyme GalNAc-T were detected [66,67]. Similarly, on microtriches of the cestode *Mesocestoides vogae* (syn. Corti), Tn and sialyl-Tn antigens were found, as well as GalNAc-T activity [68]. Cestodes also contain the Tk-antigen (GlcNAcβ1-6(GlcNAcβ1-3)Gal), which was detected in *Taenia hydatigena*, *Mesocestoides vogae* (syn. Corti) and *Taenia crassiceps* by immunoblotting using the monoclonal antibody LM389 [69].

**Figure 5:**
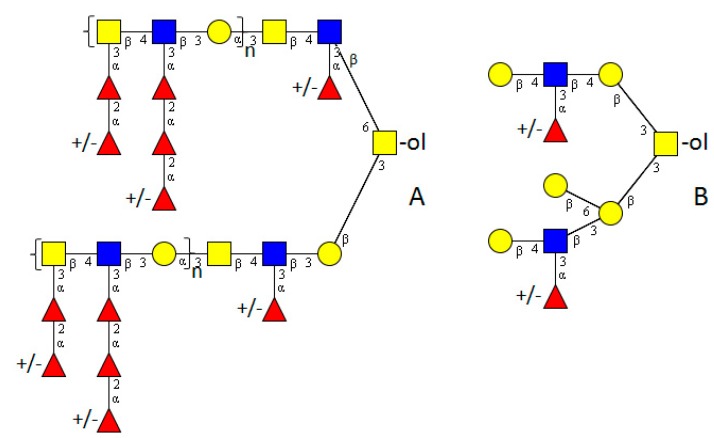
Examples of extended cercariae *O-*glycans of schistosomes. (**A**) from [70]; (**B**) from [74].

The only well-investigated representatives of platyhelminthes are trematodes of the genus *Schistosoma* (blood-fluke), which cause schistosomiasis, an acute and chronic disease. 249 million people were affected by and 42.1 million people were treated in 2012 for schistosomiasis [World Health Organization. Schistosomiasis. Fact Sheet No. 115. Geneva: available at: http://www.who.int/mediacentre/factsheets/fs115/en/ (accessed January 2015)]. The five main species, *Schistosoma mansoni*, *S. japonicum*, *S. mekongi*, *S. intercalatum* and *S.* *haematobium* are known to infect humans in south-Sahara Africa, South America, the Caribbean and the Middle and Far East. *S. mansoni*, which occurs mainly in Africa and South America, and *S. japonicum*, which occurs mainly in the Far East, were in the focus of analysis. The life-cycle of the worms takes place in different snails as intermediate hosts and is finalised in humans. Cercariae are released into water by infected snails, where they then penetrate the skin of the final host, migrate and develop into male and female adults, which mate and produce eggs. Passing from the vessels and penetrating the intestinal wall, the eggs are released with the faeces. The glycome of schistosomes is rather well investigated. Besides studies on *N*-glycans and glycosphingolipids, a number of *O-*glycan structures derived from different developmental stages are also known. In the late 1990s Khoo *et al.* identified core 1 and core 2 type *O-*glycans with further elongation by a high proportion of additional (+/−Fuc_1_)Hex_1_HexNAc_1_ units in the egg glycoproteins of *S. mansoni* and *S. japonicum*. Further, the “invertebrate core” Hex_2_HexNAc was also determined (Figure 3). *S. mansoni* and *S. japonicum* differed clearly in their fucosylation. While HexNAc-(+/−Fuc)HexNAc were terminal decoration in both schistosomes, multifucosylated termini with Fuc_1,2_HexNAc-(Fuc_1,2_)HexNAc appeared only on *S. mansoni* (Figure 5) [70]. Both egg antigens showed LacNAc (Galβ1-4GlcNAc) as well as LacdiNAc (GalNAcβ1-4GlcNAc) type structures, including Lewis^x^ (Galβ1-4(Fucα1-3)GlcNAc), as terminal elongations. Further modifications were terminal HexNAc_3_ and up to HexNAc_4_-chains directly linked to the *O-*glycan cores [70,71]. A small but important sub-fraction of *S. mansoni* egg antigens contained large heavily fucosylated branched *O-*glycan structures carrying up to four HexNAcs per chain [72]. In a recent large study on the proteome (45 proteins) of *S. mansoni,* difucosylated LacdiNAc and Lewis^x^ structures on the egg shells were detected by antibodies [73]. However, no assignment to *N*- or *O-*glycan structures has been performed.

Multifucosylated terminal sequences were seen in cercariae on core 1 and core 2 type structures with Lewis^x^, LacNAc units or single GlcNAc residues directly attached to the -3Galβ1-3GalNAc core or indirectly via a β1-6-linked Gal, forming biantennary structures (Figure 5) [74].

Other developmental stages, such as miracidia, primary sporocysts and adults, were analysed by specific antibodies for the location and structures of their glycan epitopes. The expression patterns were determined to be different in the course of the transformation process and seem to be regulated developmentally [75,76].

No genetic data exist for the corresponding enzymes; only some activity studies are available. Fuc-T activities were determined in three developmental stages (egg, cercariae and adult) of *S. mansoni,* showing a wide acceptor range. Non-sialylated core 1 type structures, core 2 type oligosaccharides with or without sialic acid or already fucosylated (such as Galβ1-4(Fucα1-3)GlcNAcβ1-3Galβ1-4Glc) were valuable acceptor substrates. Compared with the structural data, the total activity of Fuc-Ts in eggs was far higher (50 fold) than in the other investigated stages [77]. In cercariae of the schistosome *Trichobilharzia ocellata,* an α1-3-Fuc-T with broad acceptor tolerance for different variants of core 2 type oligosaccharides (unsubstituted, already α1-2-fucosylated or α1-3-sialylated) was determined. It should be noted that *N*-ethylmaleimide, which is an inhibitor of many vertebrate Lewis^x^–creating Fuc-Ts, did not inhibit the helminth enzyme. In addition, α1-2-Fuc-T activity forming difucose chains of the structure Fucα1-2Fucα1-3GlcNAc was detected [78].

The presentation of surface glycans in the different stages caused different immune responses. While adult worms did not evoke a significant immune response of the host, the eggs were highly immunogenic, which is seen in elevated Th2 response. The importance and role of the schistosome glycome in the course of host parasite interactions has been reviewed extensively [79,80]. Investigations of the different stages during the life-cycle and possible targets of interruption of this cycle have been carried out to prevent the development of the parasite or to interfere in host parasite interaction. The most common characteristic features of schistosome *O-*glycans are the terminal multifucosylated motifs, which may be promising epitopes for diagnostic antibodies.

However, preventing parasitic infection, perhaps by immunization, is only one of the medically relevant aspects of platyhelminthes. Another is to benefit from the severe reaction of the vertebrate immune system on schistosome glycans. Extracts from helminth parasites have been shown to activate the human immune system by immunomodulatory glycan structures [81]. Parasites contain minimal mucin-type *O-*glycans (T, Tn, sialyl-Tn Tk-epitopes) similar to some cancer cells, which may induce an immune response against them. These findings were supported by a negative correlation between cancer development and parasite infection (reviewed in [82]) Other studies have indicated that the T-cell response is enhanced by soluble *S. mansoni* egg antigens in general [83,84]. However, studies on mice or analysis of data of human patients showed more differentiated patterns. It is clear that there are also some immune suppressive molecules that suppress the immune response of the mammalian host during infection (e.g., in *Schistosoma* egg extract). It may be the third fucose of the multifucosylated side-chain, present on *O-*glycans of the cercarial glycocalyx, which blocks the recognition by anti-LDN-F/LDN-DR MAbs [85]. These structures could be an interesting option for a possible treatment of inflammatory diseases towards down-regulation of autoimmune or allergic responses [86], but we first must determine which of the structures correlates with which of the effects.

## 5. Mollusca

### 5.1. Cephalopoda

Two *O-*linked GalNAc residues and one *O-*linked Fuc were detected in octopus (*Octopus dofleini*) rhodopsin. *O-*glycosylation is conserved in this type of G-protein-coupled receptors; therefore, it may contribute in the maturation, transport or cell surface expression of this protein [87].

### 5.2. Gastropoda

#### 5.2.1. Bivalves

While the *N*-glycome of *Crassostrea* *virginica* (Eastern oyster) is rather complicated, the *O-*glycans found in oyster haemocytes and plasma are simple core 1 or β1-6-GlcNAc extended core 1 type structures [88]. A lectin cytochemistry study of the sorting pathways of lysosomal enzymes in *Mytilus galloprovincialis* (Mediterranean mussel) digestive cells showed that *O-*glycosylation is already fully completed in the cis-Golgi network, which is in contrast to mammals where this takes longer [3]. Although the first complete genome of a mollusc, the pacific oyster *Crassostrea gigas*, is now sequenced [89], no detailed information on its glycosylation machinery is available so far.

#### 5.2.2. Snails

**Figure 6:**
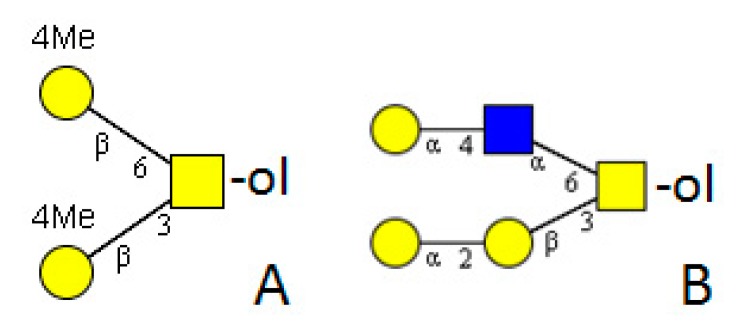
Examples of *O-*glycans from gastropod origin. (**A**) *Arion lusitanicus* [100] and (**B**) *Conus consors* [91].

Marine snails of the genus *Conus* are found in tropical areas near coral reefs. Their characteristics are beautiful shells and neurotoxic poisons which are intended for hunting and defence. Investigations of the poisons yielded the determination of several hundred, often *O-*glycosylated, peptides per *Conus* species, which may be of interest for pharmaceutical purposes (for a recent review see [90]). The focus was often on peptide analysis rather than glycans. Only a core 1 type structure was determined, while more complicated glycans were often given as Hex_2-4_HexNAc_2_, with no monosaccharide composition or linkage information. One detailed NMR study on *Conus consors* venom determined a remarkable new structure: Galα1-4GlcNAcα1-6(Galα1-2Galβ1-3)GalNAcα1-*O-*Ser (Figure 6) [91]. The unusual features of this structure are an α1-6-linked GlcNAc (usually β1-6) and terminally α-linked galactoses in l-configuration (usually β linked in d-configuration).

*Megathura crenulata* (keyhole limpet) as well as *Biomphalaria glabrata* share the structural features of their glycans with schistosomes. However, the carbohydrate epitopes have been assigned to *N*-glycans or glycolipids [92,93], or were not specified in detail [94]. A comparison of *O-*glycan structures is missing. Only for KLH 2-c, a functional unit of keyhole limpet hemocyanin, a positive binding to peanut agglutinin indicated mucin-type *O-*glycosylation [95]. Histochemical and lectin-histochemical studies on the pedal glandular system of the rayed Mediterranean limpet, *Patella caerulea*, revealed different expressions of Gal, GalNAc, α1-2- and α1-4-linked Fuc and carboxylated and/or sulphated structures, presumably in *O-*glycans, in the course of the annual cycle [96]. In some snail tissues, traces of sialic acids were identified but could not be assigned to specific glycans [97].

The most striking feature of snail glycosylation is the high degree of methylation of terminal hexose residues. 3-*O-*methyl Man, 3- and 4-*O-*methyl Gal have been identified and determined for several species of land and water snails [98,99]. The *O-*glycosylation patterns were determined for *Arion lusitanicus*, *Achatina fulica*, *Biomphalaria glabrata*, *Cepaea hortensis*, *Clea helena*, *Helix pomatia*, *Limax maximus* and *Planorbarius corneus*. The main structure was a di-methylated “invertebrate core” consisting of the protein bound GalNAc residue with two 4-*O-*methyl Gal residues in β1-3- and β1-6-position. Further elongations with Gal, Man, and Fuc in different positions were found in minor amounts (Figure 6) [100].

Thus far only, one *O-*glycosylation initiating GalNAc-T of mollusc origin has been cloned. It was a type-2 membrane protein from *Biomphalaria glabrata,* containing all domains typical for GalNAc-Ts: a short transmembrane domain, a stem region, a Gal/GalNAc motif, a manganese binding site and a ricin-like lectin domain [101]. It is homologous to other group Ib enzymes (for a classification of GalNAc-Ts see [12]) and has similar substrate specificity [102].

In *Lymnaea stagnalis*, the intermediate host for the trematode *Moliniella anceps*, a β1-4-Glc-T activity, was detected, with a substrate preference towards GlcNAc β1-6-linked to Gal or GalNAc, which may occur in *N*- as well in *O-*glycans [103]. No corresponding glycans have yet been identified.

## 6. Conclusions

All generated data show that the general glycosylation machinery is similar for all organisms, even when special features are limited to specific phyla. While core 1 type and core 2 type *O-*glycan structures are frequent modifications of vertebrates and invertebrate proteins, the “invertebrate core” has been identified only in nematodes, schistosomes and molluscs. Fuc is a common feature in terminal position of all animals, but it seems to be more dominant in invertebrates; multifucosylated and further elongated Fuc residues are possible there. Methylation of monosaccharides has never been detected in vertebrates. In invertebrates, it may occur in different linkages on Fuc or hexose residues (Gal, Man) [104]. Methylated glycans are conserved targets of animal innate defence [105], but their benefit for the invertebrate itself is still not clear. Another critical feature of glycan structures is the presence of a negative charge. This often acts as a general sorting and recognition marker. While vertebrates commonly display sialic acids in terminal position of their glycans, in invertebrates, sialic acids are very limited and clearly restricted to few tissues and/or early developmental stages. These organisms gain their negative charge by modifications with sulphate, carboxyl groups, uronic acids, aminoethylphosphate or phosphocholine.

In general, the few identified and characterised glycosyltransferases show high similarity to their mammalian counterparts in terms of structure and specificity. However, there are some important differences, such as, the ability of T-synthases of flies and nematodes to transfer Gal without needing a chaperon. We know that glycosylation is an important factor in several biological recognition processes and we know that invertebrates are extremely successful in their survival strategies and their adaption abilities to changing environments. Therefore, their glycosylation abilities seem to be an interesting target for further research in order to learn more about the influence and regulation of external factors on biosynthetic pathways in general.
